# A Systematic Review of Tripoding: A Step Towards Successful Rehabilitation

**DOI:** 10.7759/cureus.31095

**Published:** 2022-11-04

**Authors:** Rewa A Kawade, Seema Sathe, Aditee Apte, Anjali Bhoyar, Tanvi Jaiswal, Surekha A Dubey

**Affiliations:** 1 Prosthodontics, Sharad Pawar Dental College and Hospital, Datta Meghe Institute of Medical Sciences, Wardha, IND

**Keywords:** tilt of cast, cast surveyor relation, reorientation of cast, tripodization, tripoding

## Abstract

This article aims to evaluate different methods and techniques published in the literature for tripodization in removable partial dentures. The systematic review was conducted according to the Preferred Reporting Items for Systematic Reviews and Meta-Analyses (PRISMA) guidelines. Electronic databases like PubMed, Cochrane Library, and Science Direct were searched for manuscripts published till August 15, 2021. An electronic search was restricted to the English language of the publications to identify the relevant articles on tripoding techniques in removable partial dentures. A hand search was also carried out. A total of 18 articles were included in this systematic review. The documented 18 articles associated with the tripoding techniques were reviewed systematically. Accurate repositioning of a cast on the surveyor is a critical step in the fabrication of a removable prosthesis. Based on the ease of use and no modifications to the existing cast, the "tripoder attachment" and "swiveling device" can be concluded to be superior to others. Once constructed, these devices are easy to use, can be operated for various patients, do not modify or damage the cast, and can be stored and disinfected for repeated use.

## Introduction and background

Surveying the partially dentate casts is an indispensable step while fabricating a removable partial prosthesis. The surveying of models is to determine the path of insertion of the removable prosthesis. This insertion path must be decided before a prosthesis design. Casts are surveyed to determine the favorable and unfavorable undercuts that will contain the future clasp assemblies. The casts' tilt can be altered until we get a position where all undercuts are in a favorable position for fabricating the prosthesis. This tilt of the cast has to be preserved for future use; hence, tripoding is performed [1,2].

Tripoding is performed traditionally by marking the cast surface with an undercut gauge, also known as the "tripod-marks method". However, this is time-consuming and requires basic skills to perform the reorientation [3,4]. The second method is marking vertical lines on the sides of the base of the cast parallel to the surveying arm [3]. The second method will bypass the framework and reduce the chance of interference. In the third technique, tripod markings are made not on the anatomic areas but on the sides of the base of the cast [2]. However, these modalities could not sustain the model's handling while trimming, when exposed to water, and during duplication [5]. Hence another method was suggested by Wagner and Forgue [4] in which they cemented a cylindrical beveled tool in the cast. They did not interfere with the design of the prosthesis. This method provided accurate repositioning but prevented mounting and rendered the model prone to breakage.

Many proposed methods for re-establishing the cast surveyor relationship in the literature have included various devices and techniques. Therefore, the objective of this article was to evaluate, compare, and find the best possible technique among multiple strategies and procedures published in the literature for tripodization in removable partial dentures and to conduct a systematic review of the innovations made in the genre.

## Review

Materials and methods

This systematic review was conducted according to the Preferred Reporting Items for Systematic Reviews and Meta-Analyses (PRISMA) guidelines. The focus question was framed in PICO format (see Table 1).

**Table 1:** Focus question according to PICO format PICO - population, intervention, comparison, and outcome

Areas of focus	Criteria
Population	Rehabilitation using removable partial dentures.
Intervention	Use of different tripoding techniques for duplication of the tilt of a cast on the surveyor.
Comparison	Conventional tripoding techniques for reproducing the tilt of the cast.
Outcomes	
Primary outcome	The accuracy of repositioning the cast on the surveyor.
Secondary outcome	Ease of reproduction of the tilt of the cast by the dental technician and feasibility of the technique and time taken for the same.

Inclusion Criteria

Authors who have proposed tripoding techniques, including devices or methods for removable partial dentures, were included. Unpublished articles were not included in this review.

Exclusion Criteria

The techniques not proposed for tripoding of removable partial dentures and articles in languages other than English were excluded from the review.

Search Strategy

Three reviewers were involved in the process - Rewa Kawade (RK), Seema Sathe (SS), and Aditee Apte (AA). All of them searched three electronic databases (PubMed, Cochrane Library, and Science Direct) for manuscripts published until August 15, 2021. Full texts of the reviewed articles which fulfilled the inclusion criteria were obtained. The last search was done manually from the cross-references of the selected articles and citations to include all the pertinent articles. There was no disagreement between the reviewers.

**Figure 1:**
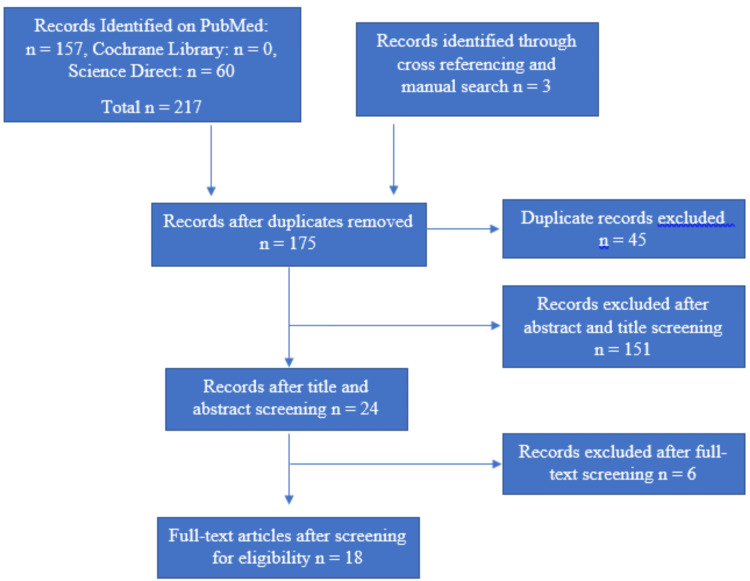
PRISMA flowchart PRISMA - Preferred Reporting Items for Systematic Reviews and Meta-Analyses

The total number of articles displayed with search terms "tripoding", "tripodization", "reorienting", "cast surveyor relation", and "tilt of cast" in the advanced search resulted in 157 articles in PubMed, zero articles in Cochrane Library and 60 articles in Science Direct. After removing duplicates, screening titles, and abstracts, 23 records were eligible. One article identified during cross-referencing of included studies was also included. A total of 24 papers were included. Five articles were found to be comparative studies; thus, they were excluded. Consequently, 18 articles were included in the analysis (Figure 1).

Results of Data Extraction

The 24 articles included for the full-text screening were obtained and studied by the reviewers, of which five articles were found to be comparative studies and hence were excluded. One article gave the technique for the fixed partial restorations and therefore was also excluded. The articles were then analyzed to check if they satisfy the six requirements of a tripoding technique. These requirements are the accuracy/dimensional stability of the device as different materials were used by the authors, simplicity of their device, ease of reorientation, applications of the device in multiple patients, if it requires cast modifications, and the ease of storage and disinfection.

Analysis of the studies

**Figure 2:**
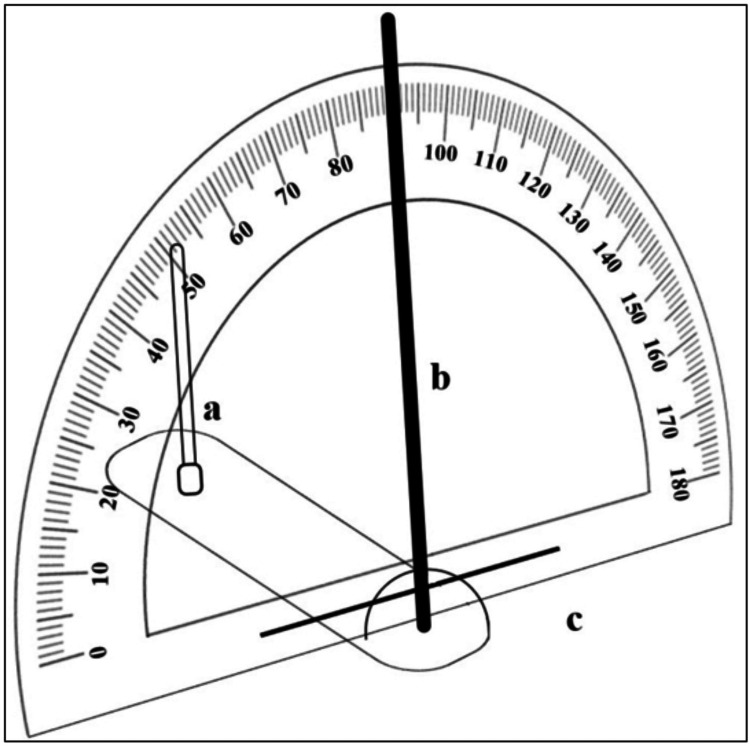
Protractor containing simplified device (a) attaches in the surveyor mandrel, the vertical rod (b) around which protractor (c) rotates.

Kaloyannides first proposed a simple device that comprised a protractor for measuring the tilt of the cast in the anteroposterior as well as transverse direction and recording the values of the same as indicated by the device (Figure 2) [3]. The dentist could note these values along with the anatomic points for further communication with the technician rendering it easy to reproduce the cast tilt.

**Figure 3:**
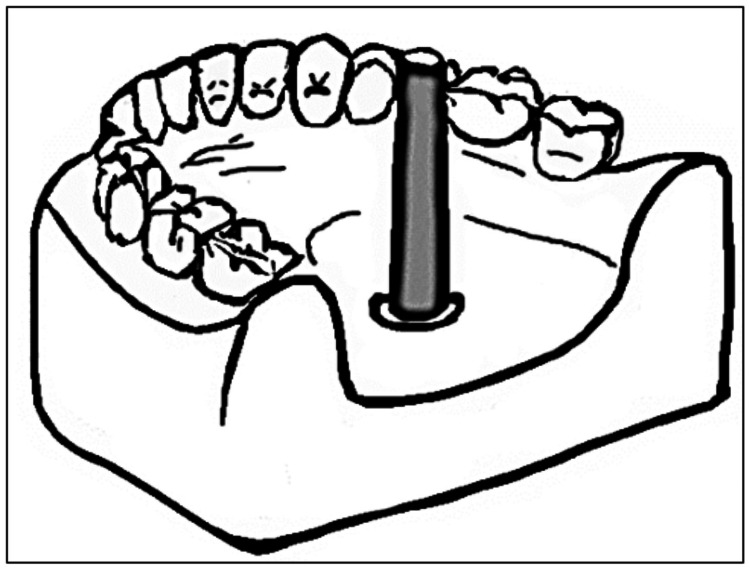
Modified cast by insertion of bur shank in lingual space of a mandibular cast

Knapp et al. modified the cemented pin method. He used a pear-shaped bur to create perforations of 4 to 6 mm diameter and 5 to 8 mm depth on the lingual space of the mandibular cast [6]. Later, the cast was surveyed conventionally, and the bur was placed in the surveying arm such that its shank acted as the analyzing rod. This assembly was now lowered inside the perforation and sealed in place using cold-cure acrylic resin (Figure 3). Reorientation of the cast only required the bur shank's insertion in the surveyor's vertical arm. The author suggested that the bur could be removed from the acrylic using pliers for mounting. 

**Figure 4:**
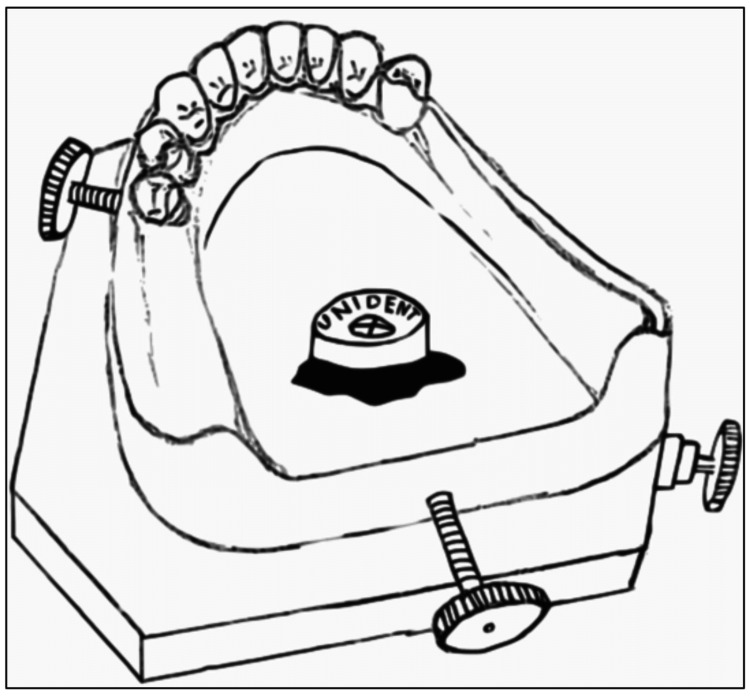
MS leveling device attached on a mandibular cast using sticky wax

Sykora stressed the disadvantage of the traditional cast indexing method of being eliminated during cast handling in the lab [5]. As the solution to the before-mentioned problem, he advocated using MS leveling device (Unident Limited, Nova Scotia). This device could be attached to the part of the cast outside the framework using what he described as "soft periphery wax". It was then adjusted so that the air bubble pointed in the center of the device's cross-marking. This assembly is fixed using sticky wax (Figure 4). The cast could be reoriented by positioning the bubble on the device again in the center.

**Figure 5:**
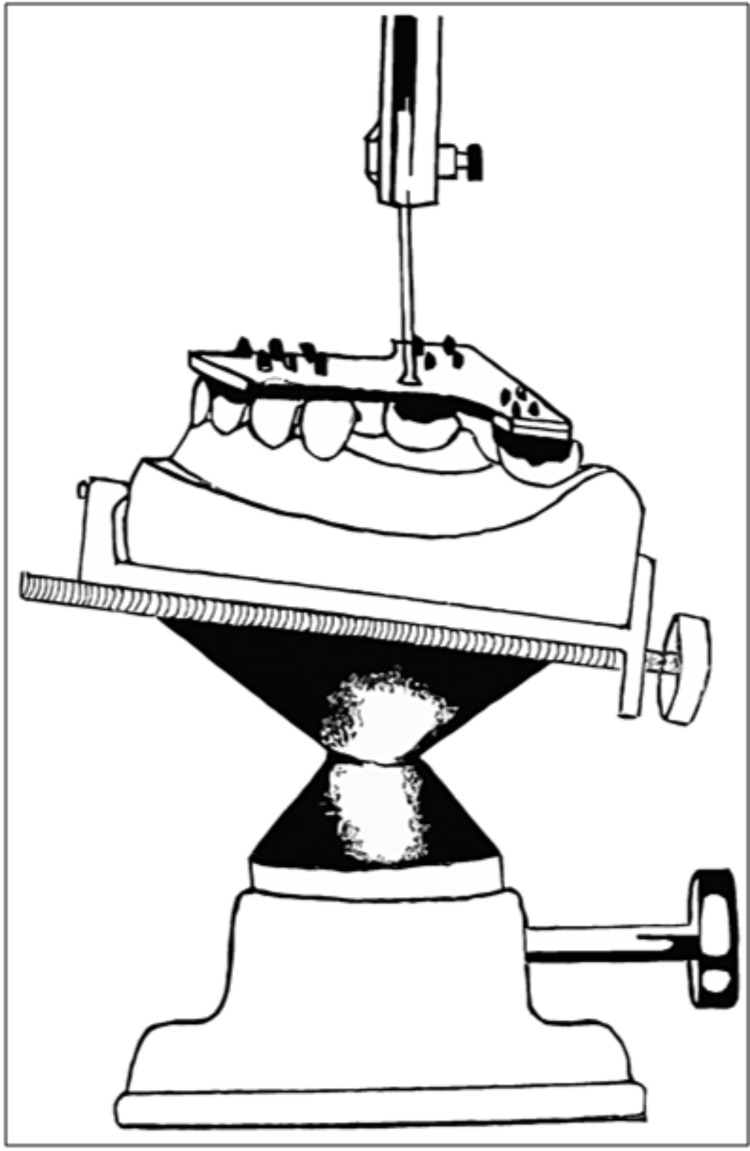
Position recorder device attached to the cast and inserted in surveyor mandrel

A new "position recorder device" was given by Sarnat and Klugman in 1981 [16]. The device consists of a three-ended plate made of plastic with two concave sides, large enough to accommodate all sizes of arches. Each end of the plate includes retentive holes corresponding to the single anterior and two posterior points used for tripoding. Besides the holes was a marking area that could note the tooth number used for marking. The plate's center had an attached mandrel handpiece that accommodated the surveying arm. Now, the cast was coated with separating media to record the tilt, and modeling wax was made to flow through the holes. Cold water was used to harden the wax. The dentist could now send the assembly to the lab. For repositioning, the device is first to fit on the cast then the handpiece mandrel is locked in the surveyor (Figure 5).

De Fiori and Miranda in 1983 described a technique including the cemented pin method [17]. First, they register the path of insertion by Wagner's way. Then they made an acrylic plate of 2 mm thickness to conform with the cast and include the reference points. The middle portion of this plate is cut to remove the interference of the cemented pin on the cast. The reference points are lubricated, and self-cure resin is utilized to record the position of the points on the acrylic plate. Now, while the cast was positioned using the cemented pin, this construction was placed on the model. Carefully the pin is removed as the surveying arm raises, and another metal pin is inserted into it. The other end of the pin is glued to the acrylic plate using self-cure resin. This "transfer guide" can now be used for other working casts of the same patient.

**Figure 6:**
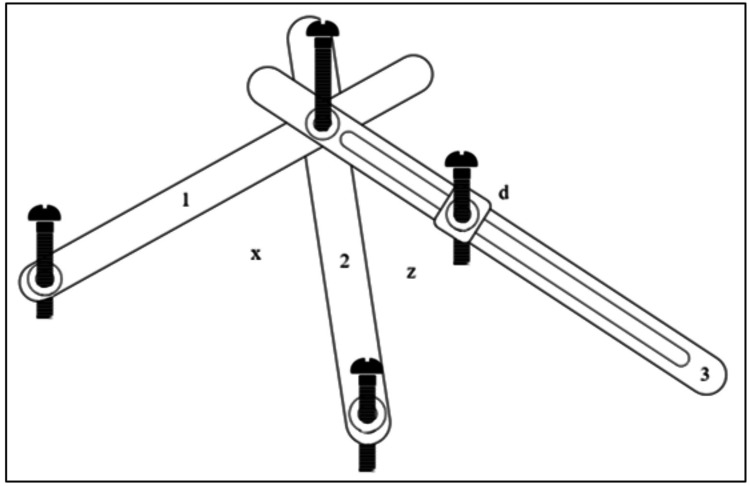
Simple recording instrument Angles X and Z may be altered. Metal strips 1 and 2 are identical. Slider (d) may be moved along metal strip 3.

Steas in 1987 fabricated a device that he named a simple recording instrument (Figure 6) using three metal strips, two short and one long [18]. The short ones were 5.5 cm, and the long one was 7.5 cm in length. The more petite strips had two holes at the two ends, and the longer one had a single hole and a central slot cut out of 4.5 cm. These strips were attached to a bur that slid easily into the surveying arm. The other end of the strips also had nuts and bolts attached. The device became fully adjustable as one could easily adjust the nuts to contact three reference points on the cast and fix them in place using the bolts. The reference points is marked on the cast for further understanding. Hence this instrument could be stored and sent to the lab and reused for other patients too. Any point on the casts could be used as anatomic landmarks for tripoding.

In 1994 Ansari produced a U-shaped plastic tray (made of tray material) by adapting modeling wax after doubling its thickness on any completely dentate cast [19]. This tray was trimmed, and its borders smoothened out. Another batch of tray material was mixed to form a bridge across the U-shaped tray, and yet another set was used to create a cylinder in the middle of the bridge to accommodate the straight stylus of the surveyor. This was then trimmed and finished. For recording the tilt, the silicone putty was manipulated and placed in the U-shaped depression. A plastic tray was then placed in the surveyor with the stylus. Lowering this assembly on the surveyed cast recorded the index of the occlusal surface of the model. After the material was set, it was separated, and the margins were trimmed to expose the cervical third of the buccal surfaces of the teeth. This could be stored and used by simply reattaching the index on the cast. This customized device could be reused by removing the putty when it was no longer needed to orient one cast.

Bezzon et al. modified the cemented pin technique in 2000 to make it possible to detach the pin from the cast [15]. They utilized a sheath and a pin that fit in it. The procedure of recording the tilt was the same as the cemented pin technique by making a slot for the sheath in the cast. After surveying, the sheath was inserted in the pin, then lowered in the perforation in the cast and fixed in place by pouring dental stone around it. This was then allowed to set, and hence a detachable pin for the model was obtained. The reorientation was easy as the pin needed to be implanted in the cast sleeve.

Dumbrigue and Chingbingyoung 2003 used a "cast angle tool (CAT)" that measured the tilt of the cast [20]. The ventral surface of the CAT was layered with vinyl polysiloxane putty material and was placed on the occlusal surface of the cast to form indentations on the putty. The CAT would indicate the value of the tilt in degrees in both sagittal and frontal planes with a preciseness of 1°. This tilt could be then noted down for each patient, and then the cast could be adjusted till the value noted is indicated in the CAT hence, reproducing the cast tilt.

**Figure 7:**
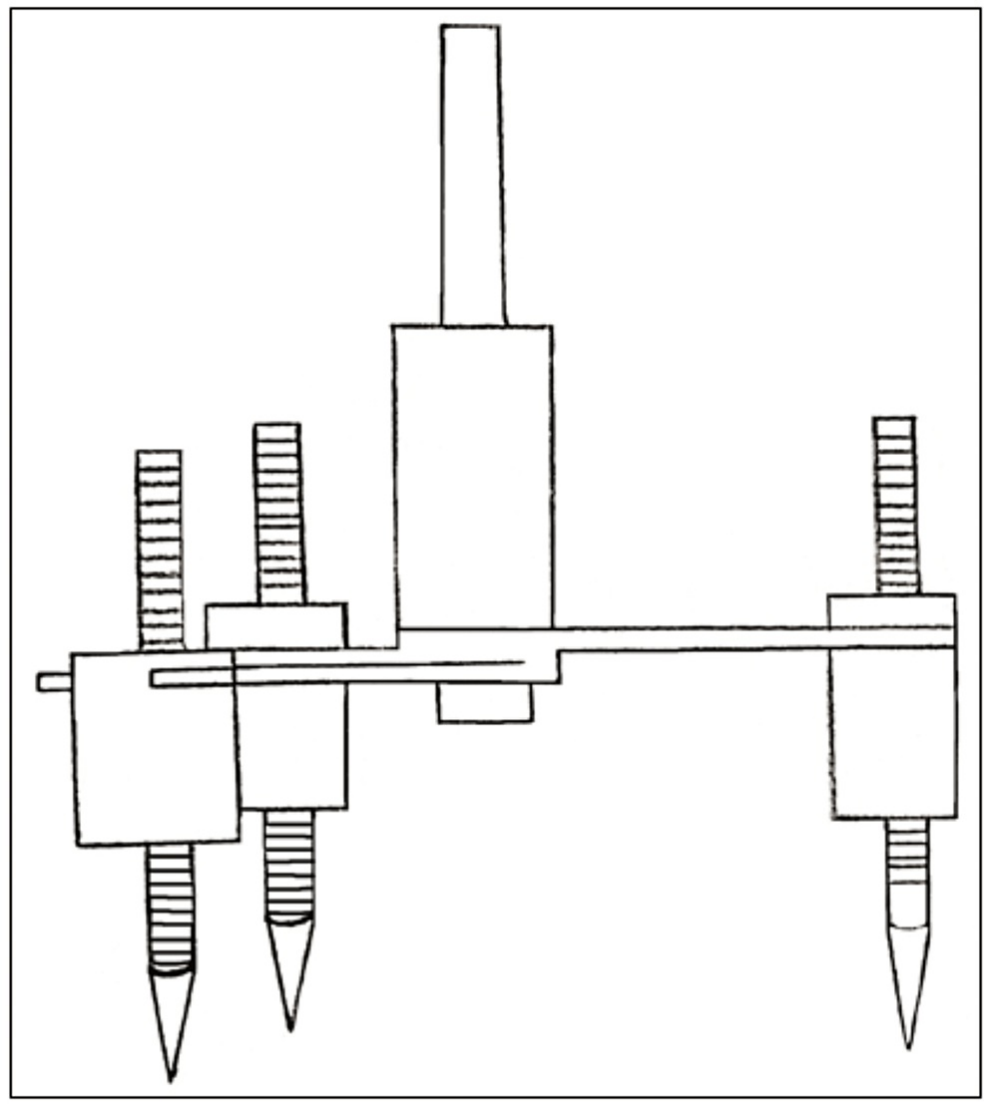
The tripoder attachment device

In 2006, Sajjan gave a "tripoder attachment" (Figure 7) that had three graduated pointers that could be vertically adjusted and sleeves that could horizontally move along the track given and locked at any height by the thumb screw [21]. Assembly affixed to the central shaft with blades that also allowed rotation around it. This shaft would then fit in the surveying arm. The height of the pointers could be changed, and the change was noted due to the presence of graduated markings. These pointers represented three reference points on the cast. The use of markings on the tool could be recorded for a cast and enabled the accurate repositioning of the graduated arms on the cast.

Kamble et al. gave an innovation in which they used a magnetic device (cobalt-samarium magnets) secured in place with two brass keepers at both ends using a nut and bolt [7]. The diameter of the magnets and brass keepers was kept the same at 30 mm. Two units of this assembly were prepared; the end of one of the bolts was ground to form a point, and in the other bolt, a hole was drilled to receive the straight handpiece mandrel. Surveying was implemented in the usual manner and a hole of 8×8×4 mm was made in the cast base. The assembly with the mandrel was then attached to the surveyor and the other end to the cast. Dental plaster was used to fix this magnet-containing assembly. This construction could be easily detached with a pull and reoriented by simply bringing the cast near a magnet.

Shakibamehr et al. gave a technique utilizing auto-polymerized acrylic resin [8]. He manipulated the resin till it reached the dough stage. He then obtained a 2 mm thickness for the resin using a glass slab. The occlusal surfaces of the teeth were lubricated using petroleum jelly, and it was placed on the resin sheet. The resin was now inscribed with the indentations of the teeth. On the opposite surface of this sheet, a bulk of resin was placed, and a lubricated analyzing rod was lowered into it. For reorientation, the indentations were matched with the cast, and the analyzing rod was placed in the index.

Kamble and Parkhedkar suggested another device containing a "dowel pin and sleeve" that was also a modified version of the cemented pin method [9]. A hole of 6×6×4 mm dimension was created in the center between the alveolar ridges such that it did not cause any interference with the framework. The dowel sleeve device was assembled and locked in the surveying mandrel. This dowel pin is then brought down to fit in the made perforation. The perforation was wetted, and type III gypsum was poured around the sleeve. After setting, the dowel pin could be removed and replaced for reorientation.

**Figure 8:**
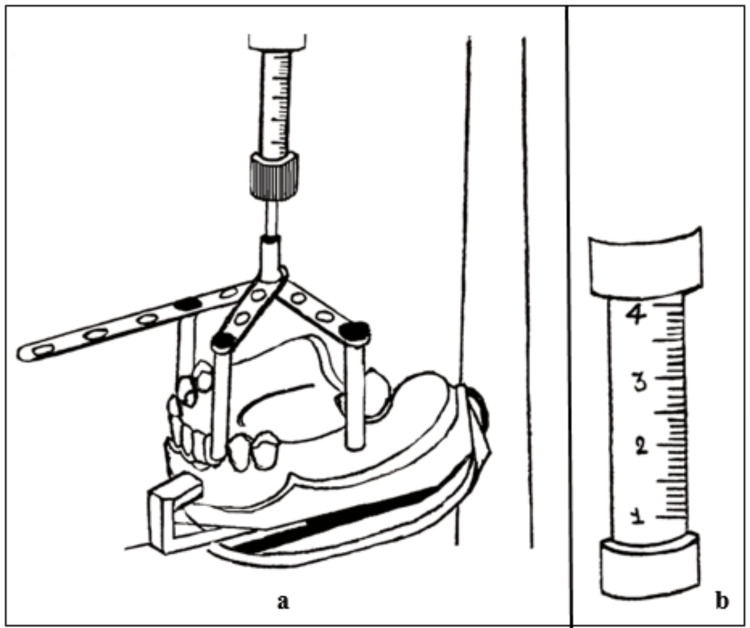
Swiveling device (a) with graded surveyor spindle (b)

Savabi and Shirban graded the surveyor spindle in millimeters till it covered the whole spindle [10]. They then fabricated a device consisting of one long strip with seven holes and two short narrow strips with five holes attached to a central handpiece mandrel. A screw and nut were attached to the end of each strip. These screws were to be adjusted in the order of the short strips first simultaneously and later the third screw in a way that they contacted three marked points on the cast (Figure 8). The surveyor spindle reading was noted, and the bolts held the screws in place.

Patil et al. in 2016 gave another method of customized registration of tilt of casts using silicone putty impression material on the surveyor tool [11]. He surveyed the cast as usual, then attached an analyzing rod to the vertical arm and moved its tip within a 5 mm range of the selected tooth that he preferred to rather be in the corner of the arch. Condensation silicone of putty consistency was mixed and adapted on the cast to cover at least five teeth extending to the marginal gingiva and 15 mm of the analyzing rod. This increases the reliance on the qualities of the imprint material for tilt reproduction accuracy.

Afsal et al. in 2017 used a worn diamond disk for the tripoding record [12]. The conventional procedure was carried out for surveying, and the worn diamond disk was placed at an adequate distance in the middle of the cast. A putty indentation was made, keeping out the anteriors and the occlusal surface of the posteriors. The index was allowed to be set after the excess was removed. For reorientation, the putty index-diamond disk construction was installed in the vertical arm. This strategy allowed for a clearer view of the index's indentations, which sped up the tripoding process. The drawback of this modification remains the same as in Dumbrigue's, Ansari's, and Patil's methods.

**Figure 9:**
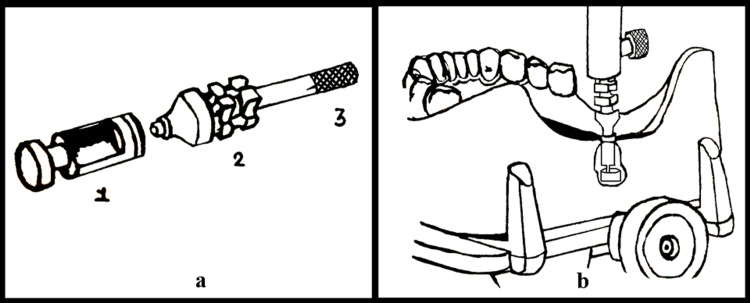
Implant impression coping assembly (a) The assembly shows 1) implant analog, 2) short, direct impression coping, 3) long retaining screw, and (b) implant coping-analog assembly in place.

Lee et al. in 2017 utilized the implant impression coping and implant analog for tripoding [13]. He selected the posterior aspect of the cast to prepare an upright groove by a tungsten carbide bur. Dimensions of the groove were made to be 10 mm deep and 20 mm long. He then connected the implant analog to a short and direct impression coping employing a long retaining screw. This retaining screw entered the vertical arm after the traditional surveying. The implant analog fit in the groove was stabilized in place using a thermoplastic adhesive (sticky wax) in a heat gun (Figure 9). The duplication of the cast was also simplified by simply replacing the direct transfer impression coping with the indirect transfer coping. This duplication was carried out using polyvinyl siloxane duplicating material after the reseating of the coping analog. This was then poured in a die stone (type IV gypsum). The reseating of the coping will transfers the cast orientation. The retaining screw goes in the surveyor's arm hence reproducing the cast tilt. This is the clearest and most straightforward of all the approaches.

**Figure 10:**
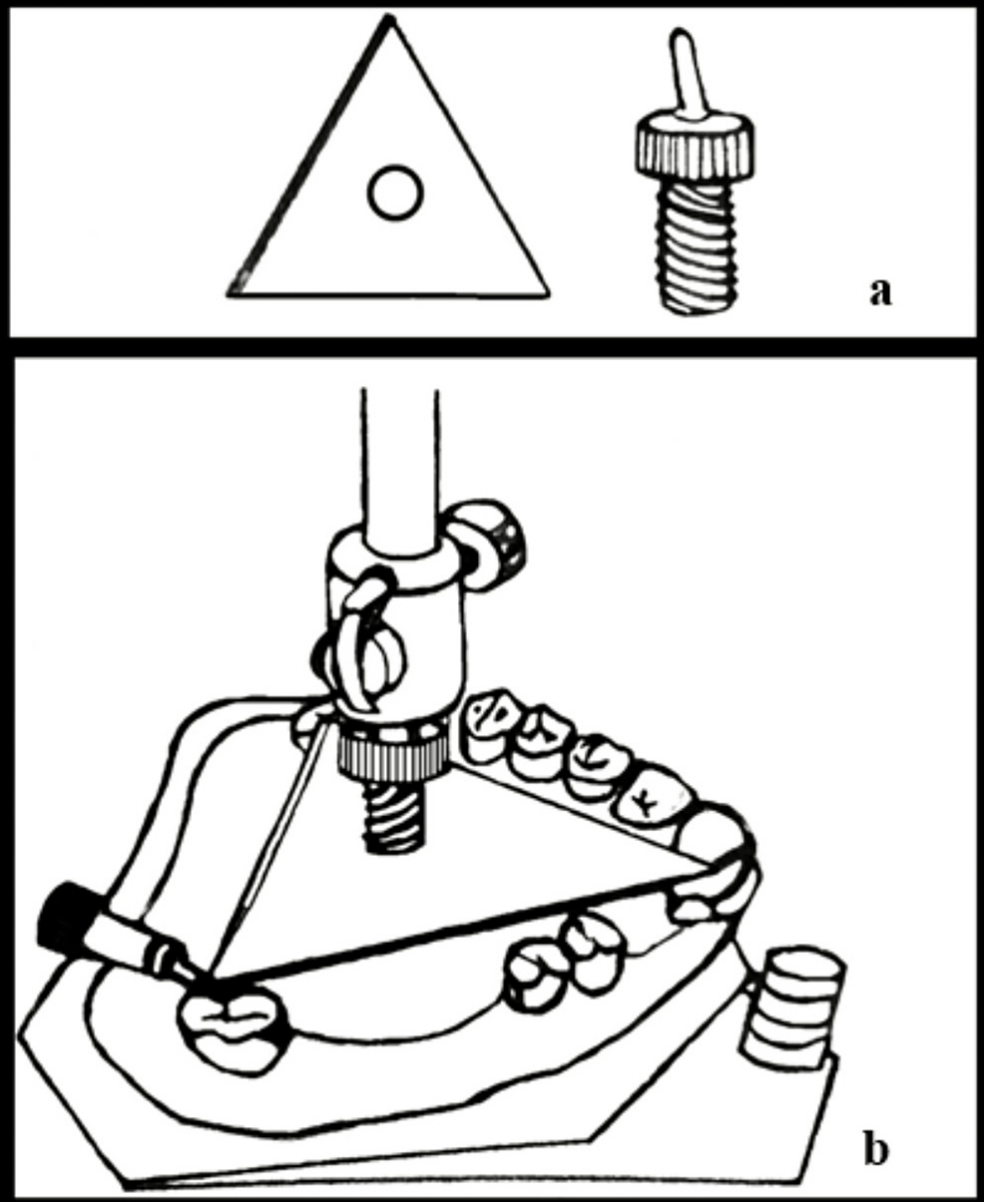
Three-point contact device (a) a triangular plate and an Allen screw, (b) a device positioned on a cast

Lastly, Gali et al. in 2019 built a "three-point contacting device" out of triangular metal plates with a 3 mm thickness and a 5 mm diameter center aperture (Figure 10) [14]. A detachable Allen screw fit in this aperture. These plates were classified into sizes of the sides of the triangle 25 mm, 30 mm, 35 mm, 40 mm, and 45 mm. These measurements are based on averages of distances measured between the incisal edges and cusp points on several casts. This enabled the accommodation of arches of various sizes. The corners of the plate will correspond to the posterior and anterior reference points, and the plate itself will form the reference plane. A lead marker marked these points on the cast. For re-registration of the tilt, the cast had to be adjusted till the three points contacted the triangle plate. Even if the construction of the device could prove to be a task, the plus points embodied in the device are noteworthy.

Tripoding of removable partial dentures is an absolute necessity for the success of the final prosthesis. The conventional tripoding methods, although convenient, have the disadvantages of not being sustainable, the requirement of skill of the operator, and consumption of time. For simplification of the same and streamlining tripoding, many authors have suggested either customized techniques or devices. All these techniques and devices proposed by the authors were to simplify the recording of the cast tilt. The literature regarding these techniques and devices was studied thoroughly, and the advantages and disadvantages were weighed and compared (Table 2). While some of them suggested a modification of the cast [6,7,9,13,15], some constructed a device for the purpose [3,5,8,10-12,14,16-21]. Lee et al. method [13], though expensive, was the most straightforward and least destructive to the cast preserving the accuracy of tripoding. A thorough theoretical analysis of these newly optimized techniques in terms of their accuracy, dimensional stability, simplicity, ease of reorientation, application in multiple patients, the modification caused in the cast, ease of disinfection, storage, and reuse was done (Table 3). The devices put forward by Savabi et al. [10] and Sajjan [21] were superior and more intelligible than others. However, no studies are comparing the accuracy and ease of these techniques. Randomized trials and comparative analysis would provide further information regarding the same.

**Table 2:** Advantages and disadvantages of devices and techniques

Author Name Year	Instrument Used	Advantages	Disadvantages
Kaloyannides 1973 [3]	Protractor containing simplified device	An advantage over the traditional method as the reproduction of cast tilt would not be affected by the shape of the cast.	It could not register concave surfaces and there was leeway for interpretational errors from the technician’s aspect.
Knapp et al. 1979 [6]	Cemented pin technique	The time and skill required for the reorientation were significantly reduced.	There are chances of breakage of the cast while making the perforation and the application in the maxillary cast could be questionable if it hinders the construction of the framework. The resultant permanent change of the diagnostic or master cast may make duplicating problematic.
Sykora 1980 [5]	MS level with underpadding of wax	Relatively easy and can exactly reproduce the original path of insertion.	Chances of the leveling device to be displaced before stabilization with sticky wax.
Sarnat et al. 1981 [16]	Triangular perforated plastic plate with soft modeling plastic	This device could be customized for each patient and stored.	The dimensional stability of both the modeling wax and the plastic plate is questionable. The material used for the plate is not exactly described.
De Fiori et al. 1983 [17]	Acrylic transfer guide	Its ability to orient multiple working casts of the same patient and ease of application.	Dimensional stability of the acrylic resin plate.
Steas 1987 [18]	Simple recording instrument	It overcame the shortcomings of the previous methods of dimensional stability and permanent modification of the cast stability of the device.	The construction of the device was an intricate process
Ansari 1994 [19]	U-shaped plastic modified impression tray with silicone putty impression material	The device could be reused by removing the putty when it was no longer needed to orient one cast. It could be stored easily for reuse.	Highly dependent again on the dimensional stability of the putty and the auto-polymerizing resin of the tray.
Bezzon et al. 2000 [15]	Cemented pin and sheath	Eliminated the disadvantages of the original method by rendering the cast useful for mounting.	The risk of cast fracture during the preparation of the perforation in the cast and the interference of the pin and sheath with the design of the major connector persists.
Dumbrigue and Chingbingyoung 2003 [20]	Cast angle tool (CAT) and vinyl polysiloxane (VPS)	Simplicity and effortlessness of the technique.	The availability of the CAT was essential in both the dental office and the laboratory for this system.
Sajjan 2006 [21]	Tripoder attachment	The ease of reorientation, reusability for different patients, and accuracy due to the presence of readings on the vertical pins.	The complex construction of the device itself.
Kamble et al. 2013 [7]	Magnetic device	Simplified the cemented pin method, this innovation provides an effortless reorientation of the cast.	The availability of magnetic assembly in the dental office is in abundance for use in multiple casts. Water and plaster may cause interference in the magnetic quality of the device. This can be carried out only if there is no interference from the design of the framework.
Shakibamehr et al. 2013 [8]	Auto-polymerized acrylic resin	The index could be customized for each patient. Not technique sensitive, giving the technique an upper hand.	The dimensional accuracy of the resin would affect the process of reorientation in this technique.
Kamble et al. 2014 [9]	Dowel pin and sleeve device	The shortcoming of the fixed nature of the cemented pin was eliminated using this method which was mounting the casts on articulators without causing interferences. The fabrication was easy owing to the easy availability of material.	Can be performed only if the design of the framework permits. Nevertheless, the risk of fracturing the cast while preparation of the cave for the dowel device persists.
Savabi et al. 2015 [10]	A swiveling device (three narrow strips attached to the mandrel and screw and nut to the other ends)	The device did not alter the cast. Could be stored and used for a variety of patients.	The device's construction, as well as the additional materials necessary, make it a time-consuming task.
Patil et al. 2016 [11]	Condensation silicone putty impression material	Customized for every patient. Ease of the technique. Does not require any specific device or tray.	Distortion may occur during reorientation if the sufficient thickness of putty is not maintained.
Afsal et al. 2017 [12]	Diamond disk and addition silicone putty elastomer	Customized for each patient. Ease of technique. The diamond disk would stabilize the putty reducing the distortion risk due to material thickness.	Chances of fracture of the disk while handling if too thin.
Lee et al. 2017 [13]	Implant impression coping and implant analog	Overcomes drawbacks of duplication incapacity and interference with the design framework. This also reduced the risk of permanently damaging or fracturing the cast while preserving the benefit of the hassle-free technique.	Only the cost of the technique could prove to be a setback.
Gali et al. 2019 [14]	Customized device	This innovation had the advantage of choosing any anatomical landmarks as the three corners of the triangular device. The dependability on the properties of the material was also eliminated.	The construction of the device could prove to be a task. The selection of the correct-sized triangle was difficult.

**Table 3:** Quality assessment of devices and techniques

Technique	Accuracy/dimensional stability	The simplicity of the device/technique	Ease of reorientation (time taken)	Application in multiple patients	No modification of cast	Ease of storage and disinfection
Kaloyannides [3]			✔	✔	✔	
Knapp et al. [6]	✔		✔			✔
Sykora [5]	✔			✔	✔	✔
Sarnat et al. [16]			✔	✔	✔	✔
De Fiori et al. [17]		✔	✔	✔	✔	✔
Steas [18]	✔		✔	✔	✔	✔
Ansari [19]		✔	✔	✔	✔	
Bezzon et al. [15]	✔	✔	✔			✔
Dumbrigue & Chinbinyung [20]	✔		✔	✔	✔	✔
Sajjan [21]	✔	✔	✔	✔	✔	✔
Kamble et al. [7]	✔		✔			
Shakibamehr et al. [8]		✔	✔		✔	
Kamble et al. [9]	✔		✔	✔		
Savabi et al. [10]	✔	✔	✔	✔	✔	✔
Patil et al. [11]		✔	✔		✔	
Afsal et al. [12]		✔	✔	✔	✔	
Lee et al. [13]	✔	✔	✔	✔		
Gali` et al. [14]	✔			✔	✔	✔

## Conclusions

Accurate repositioning of a cast on the surveyor is essential for the success of a removable partial prosthesis. The techniques and devices put forth for the purpose of simplification of tripoding were evaluated in terms of their conceptual accuracy in repositioning, as suggested by the authors. The "tripoder attachment" and "swiveling device" can be concluded to be superior to others in terms of their accuracy and ease of cast reorientation. Once constructed, these devices are easy to use, can be operated for various patients, do not modify or damage the cast, and can be stored and disinfected for repeated use.
